# How accurate are coordinate systems being used for transcranial magnetic stimulation?

**DOI:** 10.3389/fnhum.2024.1342410

**Published:** 2024-01-30

**Authors:** Maria Anabel Uehara, Natasha Jacobson, Zahra Moussavi

**Affiliations:** ^1^Department of Biomedical Engineering, University of Manitoba, Winnipeg, MB, Canada; ^2^Department of Biosystems Engineering, University of Manitoba, Winnipeg, MB, Canada; ^3^Department of Electrical and Computer Engineering, University of Manitoba, Winnipeg, MB, Canada

**Keywords:** brain stimulation, cortical excitability, neuronavigation, TMS, Talairach coordinate system

## Abstract

When applying transcranial magnetic stimulation (TMS) to the brain, it is desired to be as precise as possible to reach a target area in the brain. For that, neuronavigational system using individuals’ MRI scans were developed to guide TMS pulses delivery. All neuronavigational systems need coordinates of the target area to guide the TMS coil. Talairach coordinate system, which uses the Talairach-Tournoux atlas, is the most common system used with TMS pulses. In this study we investigated how an average Talairach coordinate from 50 healthy individuals is close to the actual location of the hand area of the primary motor cortex to investigate if that elicit a motor response in the hand; thus, investigating the fitness and accuracy of the Talairach coordinate system. We performed this experiment on six individuals (ages 61–82). When applying TMS single pulses to hand area with the given Talairach coordinate system adjusted with the MRI of each participant, three participants had involuntary twitch and three participants had no consistent physical response, as corroborated by electromyography of the abductor pollicis brevis and first dorsal interosseous muscles at the resting motor threshold intensity. Subsequently, by trial-and-error, the hand area was successfully stimulated on those three non-responder participants. The largest deviation from the Talairach coordinates was found to be 19.5 mm, measured on the surface of the cranium, between the true hand area and the mean Talairach coordinate. This finding implies that using generalized coordinates might be misleading when choosing the optimal location for brain stimulation.

## 1 Introduction

Coordinate systems for neuronavigation have become a frequently used method for localizing transcranial magnetic stimulation (TMS) pulses for a desired target area of the brain. All neuronavigational systems need the coordinates of the target area to be able to guide the TMS coil. Talairach coordinate system, derived from the atlas of a post-mortem human brain dissection, is the most common system used in neuronavigation along with TMS pulses. However, the accuracy of the selected coordinates is unknown as it relies on humans having homogenous functional neuroanatomy. Therefore, this paper aims to test if a mean Talairach coordinate for the hand area primary motor cortex (M1) can elicit an involuntary hand twitch and test its accuracy compared to the traditional trial-and-error method.

### 1.1 TMS

Transcranial magnetic stimulation (TMS) is a non-invasive brain stimulation method that uses pulsed magnetic fields over a targeted brain region. The selected stimulation site depends on the treatment protocol or to examine a specific neurological function of the brain. A common stimulation area for depression and Alzheimer’s disease treatment is the dorsolateral prefrontal cortex (DLPFC) due to its association with cognitive control and the limbic system (Avissar et al., 2017; Fitzgerald, 2021; Moussavi et al., 2021).

Repetitive TMS pulses have been used as a means of treatment for many different neurological or mental disorders such as Alzheimer’s disease (Moussavi et al., 2021), persistent post-concussion syndrome (Moussavi et al., 2019), major depression (Fitzgerald, 2021), and obsessive-compulsive disorder (Modirrousta et al., 2015). In those treatment studies, the target area of the brain is localized by either physical measurement based on 10–20 electroencephalography (EEG) system (Herwig et al., 2003) or using a neuronavigation system. While neuronavigation systems, such as Brainsight (Rogue Research Inc, 2017), promise precision within a few millimeters of the target area, the system’s accuracy is limited by the accuracy of the used reference coordinate system. In this study, coordinate system accuracy and target area localization accuracy were investigated. The motor cortex is one of the only stimulation sites that enable the evaluation of whether TMS pulses induce neuron excitation. Thus, localization accuracy for the hand area was investigated by comparing trial-and-error and neuronavigation with Talairach coordinate system.

### 1.2 RMT

The resting motor threshold (RMT) is the minimum intensity of the TMS machine that is needed to evoke an involuntary motor response in an individual. RMT is determined using single TMS pulses applied over the hand area of the M1 when the hand is at rest. The two methods of determining RMT response from the TMS pulses are: (1) physical response, and (2) electromyography (EMG) signal analysis; though, it is always recommended to also use visual response in addition to EMG analysis. Visual inspection of physical response requires the administrator of TMS to observe the hand for an involuntary twitch following every pulse. Using this method, the definition of RMT is the minimum stimulus intensity required to evoke a visible motor response of the hand at rest for more than 50% of pulses (Greenberg et al., 1997). The second method uses EMG data from the first dorsal interosseous (FDI) muscle or abductor pollicis brevis (APB). RMT is defined by this method as the minimum intensity required to evoke a peak-to-peak motor evoked potential of 50 μV in 5 out of 10 trials (Borckardt et al., 2006). Assuming that the neurons excitability is the same across the neocortex, RMT is used as an individual’s minimum magnetic field intensity to apply TMS to anywhere over the cortex. Although some researchers have questioned such homogeneity in the neuronal threshold of excitation in different parts of the brain (Stokes et al., 2007), it is common practice to apply TMS pulses at 80–120% of the RMT for treatment or other neuroscience investigation (Peterson et al., 2011; Rutherford et al., 2015; Turi et al., 2021).

### 1.3 Stimulation site localization

Trial-and-error is the most used technique to determine RMT. The TMS coil is placed over the estimated motor cortex area to observe an involuntary hand twitch to confirm target acquisition. Despite its preferential use, trial-and-error is only successful if practiced by experienced researchers. These researchers must define the starting point of localizing the M1 and subsequently manoeuvre the coil’s position and angle while the coil center is tangential and in contact with the participant’s scalp. RMT tests apply single TMS pulses over the left or right M1. The location that produces the largest response in the relevant hand is defined as the “hotspot.” However, this technique is restricted to the motor cortex, as other brain areas do not have a behavioral or signature output to confirm stimulation site accuracy.

Another strategic method to localize brain stimulation sites is imaging-based navigation [magnetic resonance imaging (MRI), functional MRI, or EEG]. This technique selects a desired brain structure on a person’s MRI to determine the location to stimulate (Brett et al., 2002). Consequently, MRI-based and fMRI-based navigations are limited to the accuracy of the used coordinates and the degree of researchers’ expertise in brain anatomy, respectively. For example, precentral motor hand area localization relies on familiarity with the cerebral gyrus shape. Research has shown that the M1 hand area can be identified in an individual’s MRI as the “hand knob,” an omega or epsilon shape along the motor cortex (Yousry et al., 1997). Imaging-based navigation utilizes neuronavigation for precise navigation of the TMS coil over the skull to target specific brain regions. However, one should note that the accuracy of a neuronavigation system is proportional to the accuracy of the given coordinates of the desired target area to the software (Caulfield et al., 2022). Therefore, the question would be how accurate the coordinate of the target area is.

The International 10–20 EEG system estimates the targeted cerebral region by measuring the scalp, creating a grid, and using references such as the inion, nasion, and preauricular points (Rotenberg et al., 2014). Similar to this EEG measurement estimation, the “5 cm rule” developed by George et al. (1995), was created to target the DLPFC by measuring 5 cm anterior to the M1 hand area. This was developed in consultation with the Talairach atlas to quickly target a commonly used treatment sight. Furthermore, the Beam-F3, is another method to easily target DLPFC for TMS treatment administration which follows the International 10–20 system with less measurements (Beam et al., 2009). This method has been shown to be more precise and reliable than the 5 cm rule (Trapp et al., 2020); however, a recent study pointed out the assumptions used by the Beam-F3 method which produces an error due to head shape (Fabregat-Sanjuan et al., 2022).

For all brain localization methods, there is a trade-off between accuracy and cost (in time and money) except for the trial-and-error method. However, trial-and-error has limited application due to its requirement for direct measurable response.

### 1.4 Coordinate systems

Coordinate systems for the human brain are used to document the location of the brain independent of the size and shape of an individual brain. MRIs are transformed into a specific standard sized template brain for a common coordinate space. Transformation of an individual’s MRI to a template brain is through the alignment of the anterior commissure (AC) and the posterior commissure (PC), which indicates the x-, y-, and z-axis. The individual’s brain is scaled to match the template brain by scaling the distance between the AC and PC and all brain quadrants’ size (Brett et al., 2002). The most widely used spaces are the Talairach-Tournoux atlas, generated from a single dissected brain and the Montreal Neurological Institute (MNI) template, created from averaging multiple MRIs. This study will focus on testing the Talairach coordinates.

Previous research compared four TMS coil positioning strategies by analyzing the cognitive effects (Sack et al., 2009). The four positioning strategies are fMRI-guided TMS neuronavigation, MRI-guided TMS neuronavigation, group functional Talairach coordinates, and 10–20 EEG position P4. They concluded that Talairach coordinates had reduced effect sizes compared to fMRI-guided and MRI-guided neuronavigation approaches due to the consideration of interindividual variance, thereby improving TMS coil targeting (Sack et al., 2009).

Another study compared Talairach coordinates with the 5 cm rule to target the DLPFC (Fitzgerald et al., 2009). The study determined the mean distance of the motor cortex to the suggested Talairach coordinate was 91.81 mm (Fitzgerald et al., 2009). Therefore, the Talairach coordinate is significantly farther away from the motor cortex than 5 cm, depicting the inaccuracy of using simple EEG measurement method. It is important to note that the Talairach-Tournoux system was considered to provide accurate positioning to DLPFC location.

We investigated whether the mean Talairach coordinate can stimulate the FDI or APB of the M1 of individuals using TMS. The mean Talairach coordinates derived from 50 right-handed healthy individuals is reported by Mylius et al. (2013), as [−35.45, −26.12, 67.00].

As for testing the MNI coordinates, it was difficult to find an MNI coordinate to test. The majority of studies report the coordinate deeper in the cortex from fMRI studies that do not represent the TMS pulse localization coordinates that are used in TMS studies. Other studies have reported Talairach coordinates that were converted to MNI coordinates which in theory are the same location and would not be a different test as that for Talairach coordinate. Nevertheless, we determined the true hand area for each participant using trial-and-error method and reported both Talairach and MNI coordinates from the Brainsight TMS neuronavigation system.

## 2 Method

**FIGURE 1:**
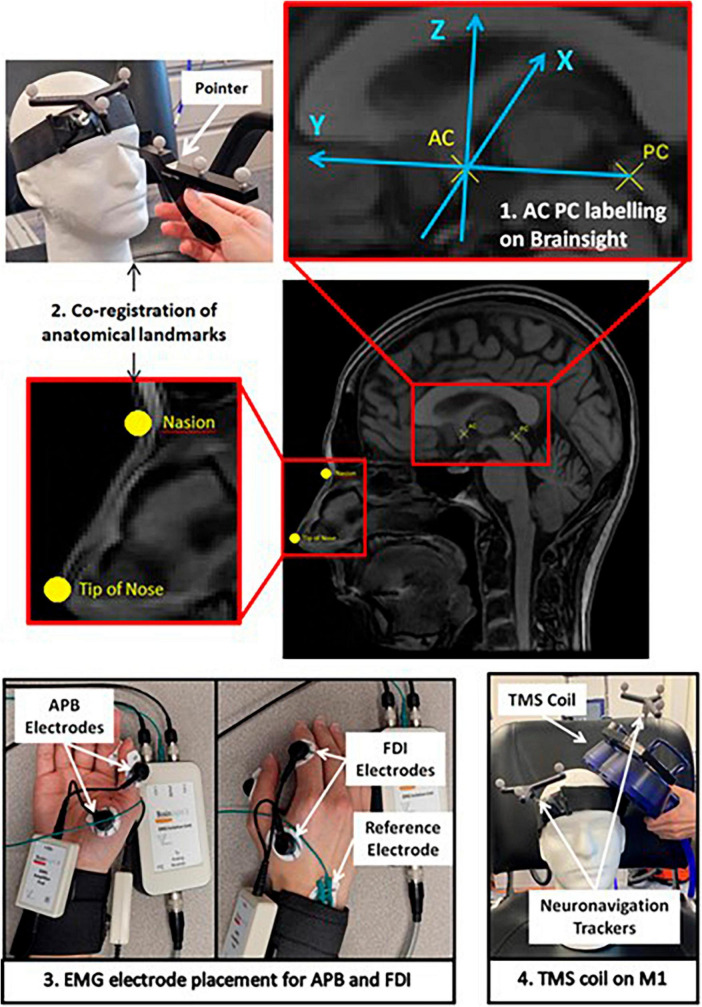
Schematic of the study protocol depicting the Brainsight software setup of participants’ MRI, EMG electrode placement on APB and FDI muscles, and demonstration of Magstim TMS coil placement on the motor cortex.

We collected data from five older adults from a TMS study for Alzheimer’s disease (Moussavi et al., 2021), and one healthy individual (62 years old). The participant demographics can be found in Table 1. T1-weighted MRIs from all participants were uploaded to the Rogue Research Brainsight TMS neuronavigation system. The MRI configuration on Brainsight, as shown in Figure 1, includes linear transformation, anatomical landmark registration, and Talairach coordinate setup. Linear transformation of the MRI to standardized space was performed by indication of AC and PC and ensuring the boundaries of the brain are properly identified for scaling. Anatomical landmarks including nasion (bridge of the nose), tip of the nose, right and left preauricular points were indicated on the automatic skin reconstruction. The final setup of the MRI was to register the mean Talairach coordinate of hand area M1 derived from 50 right-handed healthy individuals [−35.45, −26.12, 67.00] to each participant’s MRI (Mylius et al., 2013). This study was approved by the University of Manitoba Biomedical Research Ethics Board as a sub-study under HS19998 (B2016:077). All participants signed an informed consent form prior to their participation.

**TABLE 1:** Participant demographics.

Characteristic	Mean (standard deviation)
Age	72 (9.3)
Sex (male/female)	(4/2)
Resting Motor Threshold Values	52 (5.8)
Handedness (right/left)	(6/0)

For each participant, the mean Talairach coordinate was tested first, and then by trial-and-error, the true “hotspot” was determined. To begin the session, a headband with neuronavigation tracker was placed on the participant. Co-registration of the participants to their MRI was performed with a pointer indicating the physical location of the anatomical landmarks. EMG electrodes were placed on the FDI and APB muscle, the two most used muscles for RMT measurements (Sondergaard et al., 2021), to corroborate the findings from visual response when performing RMT measurement. Brainsight2 EMG pods were used to collect the EMG data using Kendall disposable surface EMG/ECG/EKG electrodes (24 mm, Ag/AgCl). Through a trigger cable between Magstim TMS and Brainsight computer system, the EMG signals were displayed on Brainsight software for a period of time around each TMS pulse. The Rapid2 Magstim Stimulator on single TMS pulse setting with the figure-eight AirFilm Coil was used to determine RMT. Subsequently, the participant received single TMS pulses 5 to 10 s apart targeted to the pre-registered coordinates by Brainsight’s visual feedback on the coil position. Secondly, the trial-and-error method was used to determine the true “hotspot” for the M1 performed by an experienced TMS user. The intensity is reduced to the minimum intensity required to produce an involuntary visible response verified with FDI and APB EMG signals. The Brainsight system records the location of every pulse. Therefore, for the trial-and-error methods the coordinates for the M1 of the thumb in both MNI and Talairach coordinate systems were determined for the participant.

## 3 Results

Using the mean Talairach coordinate [−35.45, −26.12, 67.00] to stimulate the right hand, no consistent physical response was observed in 50% of the participants at their RMT intensity. For one participant, no response was detected in the electromyography data from both FDI and APB muscles at 10% higher than the known RMT. For two participants, 50% of the pulses had an observable response at 10% higher than the known RMT. The remaining three participants, the Talairach coordinate provided the hotspot for the hand area.

**FIGURE 2:**
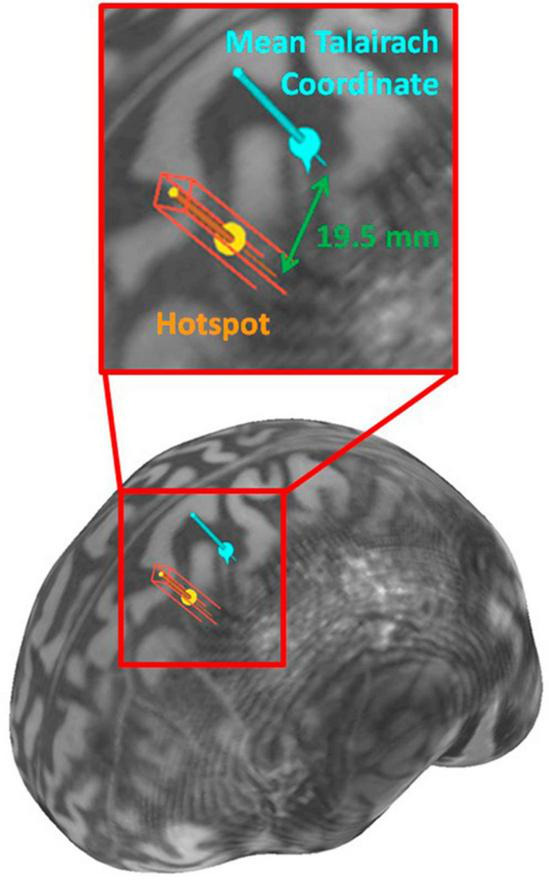
Brain surface visualization rendered from Participant 1’s MRI with the hotspot and the mean Talairach coordinate.

The accurate Talairach and MNI coordinates shown in Table 2 were determined using the trial-and-error method for the three participants with no consistent response at the mean Talairach coordinate. The scalp distance between the “hotspot” for RMT and the mean Talairach coordinate, [−35.45, −26.12, 67.00] (Mylius et al., 2013), are shown in Table 2 with the maximum being displayed in Figure 2. The mean scalp distance between the hotspot and the mean Talairach coordinate for our sample is 6.1 mm (SD = 8.1).

**TABLE 2:** Hotspot coordinates for participants with no consistent response at the mean Talairach coordinate.

Participant	Talairach coordinates	MNI coordinates	Scalp distance^a^
1	[−47.01, −8.07, 69.70]	[−48.54, −1.70, 74.36]	19.5 mm
2	[−39.85, −15.77, 65.99]	[−40.90, −10.11, 70.85]	12.3 mm
3	[−39.88, −21.64, 75.06]	[−40.85, −15.54, 81.41]	5 mm

^a^Distance between participant’s hotspot and mean Talairach coordinate [−35.45, −26.12, 67.00].

## 4 Discussion

In this study, the M1 was the selected stimulation site to measure a direct response from TMS to assess localization accuracy. Findings indicated that the mean Talairach coordinate could target the hand area of the M1 for 50% of the participants as evidenced by insufficient EMG signals on two muscles, and a lack of physical response. From trial-and-error, the largest deviation of the “hotspot” was 19.5 mm from the mean Talairach coordinates.

Three key factors to explain the result of this study are (1) the high interindividual variability in the brain functional localization, (2) the sample size used to determine the average coordinate, and (3) the sample size of this study. Using normalized MRIs to a standard template does not guarantee that functional locations of the brain are normalized, but only that the size of the brain is normalized. Therefore, the high interindividual variability that exists in the brain structure must be considered (Rademacher et al., 2001). Secondly, only 50 people were used to determine the mean Talairach coordinate. Thus, it is possible that a larger sample size could improve coordinate accuracy, though the concern of heterogeneous brain structures persists. Our limited sample size of 6 participants does not provide enough data to determine whether generalized coordinates should be used on majority of people. One solution would be to use a much larger dataset at different age groups and dementia severity to provide a better reference coordinates for different parts of the brain.

Dorsolateral prefrontal cortex is the most common site for TMS treatment of many neurological disorders including depression, Alzheimer’s disease, obsessive compulsive disorder, and more. There are contradictory results seen in literature whether the left, right, or both DLPFC are effective treatment locations. One concern in the prefrontal cortex is the variability in sulcal morphology, making it difficult to select an accurate anatomical location for all participants (Rademacher et al., 2001; Brett et al., 2002). Furthermore, it has been shown that the “hand knob” shape within the M1 presents high interindividual variability (White et al., 1997). Therefore, using the localization method based on functional anatomical knowledge may not be reliable.

Another consideration for TMS localization is the error margin that stimulates the targeted region. The action potentials that occur due to the TMS pulse affect the neurons at the cortex of the targeted region and the surrounding neurons. One research paper has suggested knowledge gaps in critical stimulation site accuracy for effective treatment (Fitzgerald, 2021). Furthermore, weaker magnetic fields have been shown to affect treatment outcomes (Opitz et al., 2015; Chow, 2019) which may contribute to the error margin for localization.

Numerous studies have used MNI or Talairach coordinates to determine the stimulation site (Fitzgerald et al., 2009; Hoffman et al., 2013; Kraft et al., 2015; Masina et al., 2018). A common practice for TMS studies is to use coordinates from previous studies based on Brodmann’s area or the mean MNI or Talairach coordinates from functional studies as the treatment site. The coordinates in standard space do not consider all the variability between people in the functional locations. This can affect treatment effectiveness and may shed light on inconsistencies in literature.

A few papers discussed the necessity of fMRI to accurately determine the stimulation site (Boroojerdi et al., 1999; Sack et al., 2009). It is suggested that the inaccurate stimulation site from methods such as mean coordinates, EEG measurements, or the 5 cm rule may be the reason for inconsistent results from TMS studies. However, fMRI is a very costly solution that is not accessible to all, and different methods should be further studied to improve accuracy.

In conclusion, using mean Talairach coordinates for TMS might not stimulate the targeted neurons for a particular treatment. This study determined that the mean Talairach coordinate [−35.45, −26.12, 67.00] for the left M1 hand area had a maximum error of 19.5 mm from the true hand area for 6 participants. The coordinate system was created to compare brain sites between people and localize findings of anatomical or functional studies. Researchers commonly use these coordinates to target specific sites, but their study design needs to consider localization accuracy. Furthermore, fMRI studies may provide improved location accuracy with the drawback of the cost in both time and money. Future research may determine improved and accurate targeting systems. Alternatively, establishing an appropriate error margin of localization accuracy may allow researchers to use MNI or Talairach coordinates to estimate the stimulation site for a specific region such as the DLPFC. This study emphasized the importance of understanding the coordinate system for brain imaging and its dependency on homogeneity of human brains.

## Data availability statement

The raw data supporting the conclusions of this article will be made available by the authors, without undue reservation.

## Ethics statement

The studies involving humans were approved by the University of Manitoba Biomedical Research Ethics Board. The studies were conducted in accordance with the local legislation and institutional requirements. Written informed consent for participation in this study was provided by the participants’ legal guardians/next of kin.

## Author contributions

MU: Conceptualization, Data curation, Formal analysis, Investigation, Methodology, Project administration, Writing—original draft, Writing—review and editing. NJ: Writing—review and editing. ZM: Funding acquisition, Resources, Supervision, Writing—review and editing.

## References

[B1] Avissar M. Powell F. Ilieva I. Respino M. Gunning F. M. Liston C. (2017). Functional connectivity of the left DLPFC to striatum predicts treatment response of depression to TMS. *Brain Stimul.* 10 919–925. 10.1016/j.brs.2017.07.002 28747260 PMC5568496

[B2] Beam W. Borckardt J. J. Reeves S. T. George M. S. (2009). An efficient and accurate new method for locating the F3 position for prefrontal TMS applications. *Brain Stimul.* 2 50–54. 10.1016/j.brs.2008.09.006 20539835 PMC2882797

[B3] Borckardt J. J. Nahas Z. Koola J. George M. S. (2006). Estimating resting motor thresholds in transcranial magnetic stimulation research and practice: a computer simulation evaluation of best methods. *J. ECT* 22 169–175. 10.1097/01.yct.0000235923.52741.72 16957531

[B4] Boroojerdi B. Foltys H. Krings T. Spetzger U. Thron A. Töpper R. (1999). Localization of the motor hand area using transcranial magnetic stimulation and functional magnetic resonance imaging. *Clin. Neurophysiol.* 110 699–704. 10.1016/S1388-2457(98)00027-3 10378741

[B5] Brett M. Johnsrude I. S. Owen A. M. (2002). The problem of functional localization in the human brain. *Nat. Rev. Neurosci.* 3 243–249. 10.1038/nrn756 11994756

[B6] Caulfield K. A. Fleischmann H. H. Cox C. E. Wolf J. P. George M. S. McTeague L. M. (2022). Neuronavigation maximizes accuracy and precision in TMS positioning: evidence from 11,230 distance, angle, and electric field modeling measurements. *Brain Stimul.* 15 1192–1205. 10.1016/j.brs.2022.08.013 36031059 PMC10026380

[B7] Chow Z. R. (2019). Sham treatment is as effective for treatment-resistant depression as repetitive transcranial magnetic stimulation. *JAMA Psychiatry* 76:99. 10.1001/jamapsychiatry.2018.2755 30422254

[B8] Fabregat-Sanjuan A. Pàmies-Vilà R. Pascual-Rubio V. (2022). Evaluation of the beam-F3 method for locating the F3 position from the 10–20 international system. *Brain Stimul.* 15 1011–1012. 10.1016/j.brs.2022.07.002 35863653

[B9] Fitzgerald P. B. (2021). Targeting repetitive transcranial magnetic stimulation in depression: do we really know what we are stimulating and how best to do it? *Brain Stimul.* 14 730–736. 10.1016/j.brs.2021.04.018 33940242

[B10] Fitzgerald P. B. Maller J. J. Hoy K. E. Thomson R. Daskalakis Z. J. (2009). Exploring the optimal site for the localization of dorsolateral prefrontal cortex in brain stimulation experiments. *Brain Stimul.* 2 234–237. 10.1016/j.brs.2009.03.002 20633422

[B11] George M. S. Wassermann E. M. Williams W. A. Callahan A. Ketter T. A. Basser P. (1995). Daily repetitive transcranial magnetic stimulation (RTMS) improves mood in depression. *Neuroreport* 6 1853–1856. 8547583 10.1097/00001756-199510020-00008

[B12] Greenberg B. D. George M. S. Martin J. D. Benjamin J. Schlaepfer T. E. Altemus M. (1997). Effect of prefrontal repetitive transcranial magnetic stimulation in obsessive-compulsive disorder: a preliminary study. *Am. J. Psychiatry* 154 867–869. 10.1176/ajp.154.6.867 9167520

[B13] Herwig U. Satrapi P. Schönfeldt-Lecuona C. (2003). Using the international 10-20 EEG system for positioning of transcranial magnetic stimulation. *Brain Topogr.* 16 95–99. 10.1023/B:BRAT.0000006333.93597.9d 14977202

[B14] Hoffman R. E. Wu K. Pittman B. Cahill J. D. Hawkins K. A. Fernandez T. (2013). Transcranial magnetic stimulation of Wernicke’s and right homologous sites to curtail voices: a randomized trial. *Biol. Psychiatry* 73 1008–1014. 10.1016/j.biopsych.2013.01.016 23485015 PMC3641174

[B15] Kraft A. Dyrholm M. Kehrer S. Kaufmann C. Bruening J. Kathmann N. (2015). Brain stimulation TMS over the right precuneus reduces the bilateral field advantage in visual short term memory capacity. *Brain Stimul.* 8 216–223. 10.1016/j.brs.2014.11.004 25481073

[B16] Masina F. Vallesi A. Di Rosa E. Semenzato L. Mapelli D. (2018). Possible role of dorsolateral prefrontal cortex in error awareness: single-pulse TMS evidence. *Front. Neurosci.* 12:179. 10.3389/fnins.2018.00179 29618969 PMC5871703

[B17] Modirrousta M. Shams E. Katz C. Mansouri B. Moussavi Z. Sareen J. (2015). The efficacy of deep repetitive transcranial magnetic stimulation over the medial prefrontal cortex in obsessive compulsive disorder: results from an open-label study. *Depress. Anxiety* 32 445–450. 10.1002/da.22363 25826717

[B18] Moussavi Z. Rutherford G. Lithgow B. Millikin C. Modirrousta M. Mansouri B. (2021). Repeated transcranial magnetic stimulation for improving cognition in patients with Alzheimer disease: protocol for a randomized, double-blind, placebo-controlled trial. *JMIR Res. Protoc*. 10:e25144. 10.2196/25144 33416500 PMC7822717

[B19] Moussavi Z. Suleiman A. Rutherford G. Pouya O. R. Dastgheib Z. Zhang W. (2019). A pilot randomised double-blind study of the tolerability and efficacy of repetitive transcranial magnetic stimulation on persistent post-concussion syndrome. *Sci. Rep.* 9:5498. 10.1038/s41598-019-41923-6 30940870 PMC6445141

[B20] Mylius V. Ayache S. S. Ahdab R. Farhat W. H. Zouari H. G. Belke M. (2013). Definition of DLPFC and M1 according to anatomical landmarks for navigated brain stimulation: inter-rater reliability, accuracy, and influence of gender and age. *Neuroimage* 78 224–232. 10.1016/j.neuroimage.2013.03.061 23567888

[B21] Opitz A. Legon W. Mueller J. Barbour A. Paulus W. Tyler W. J. (2015). Is sham CTBS real CTBS? The effect on EEG dynamics. *Front. Hum. Neurosci.* 8:1043. 10.3389/fnhum.2014.01043 25620925 PMC4287020

[B22] Peterson K. McCleery E. Waldrip K. (2011). *Evidence brief: factors that optimize therapy with repetitive transcranial magnetic stimulation for treatment-resistant depression. VA evidence-based synthesis program evidence briefs.* Washington, DC: Department of Veterans Affairs. 27606395

[B23] Rademacher J. Bürgel U. Geyer S. Schormann T. Schleicher A. Freund H. J. (2001). Variability and asymmetry in the human precentral motor system: a cytoarchitectonic and myeloarchitectonic brain mapping study. *Brain* 124 2232–2258. 10.1093/brain/124.11.2232 11673325

[B24] Rogue Research Inc (2017). *Brainsight user manual.* Montréal, QC: Rogue Research Inc.

[B25] Rotenberg A. Horvath J. C. Pascual-Leone A. (2014). *Transcranial magnetic stimulation*, Vol. 89. New York, NY: Springer. 10.1007/978-1-4939-0879-0_17

[B26] Rutherford G. Lithgow B. Moussavi Z. (2015). Short and long-term effects of RTMS treatment on Alzheimer’s disease at different stages: a pilot study. *J. Exp. Neurosci.* 9 43–51. 10.4137/JEN.S24004 26064066 PMC4457230

[B27] Sack A. T. Cohen Kadosh R. Schuhmann T. Moerel M. Walsh V. Goebel R. (2009). Optimizing functional accuracy of TMS in cognitive studies: a comparison of methods. *J. Cogn. Neurosci.* 21 207–221. 10.1162/jocn.2009.21126 18823235

[B28] Sondergaard R. E. Martino D. Kiss Z. H. T. Condliffe E. G. (2021). TMS motor mapping methodology and reliability: a structured review. *Front. Neurosci*. 15:709368. 10.3389/fnins.2021.709368 34489629 PMC8417420

[B29] Stokes M. G. Chambers C. D. Gould I. C. English T. McNaught E. McDonald O. (2007). Distance-adjusted motor threshold for transcranial magnetic stimulation. *Clin. Neurophysiol.* 118 1617–1625. 10.1016/j.clinph.2007.04.004 17524764

[B30] Trapp N. T. Bruss J. King Johnson M. Uitermarkt B. D. Garrett L. Heinzerling A. (2020). Reliability of targeting methods in TMS for depression: beam F3 vs. 5.5 cm. *Brain Stimul.* 13 578–581. 32289680 10.1016/j.brs.2020.01.010 PMC7507589

[B31] Turi Z. Lenz M. Paulus W. Mittner M. Vlachos A. (2021). Selecting stimulation intensity in repetitive transcranial magnetic stimulation studies: a systematic review between 1991 and 2020. *Eur. J. Neurosci.* 53 3404–3415. 10.1111/ejn.15195 33754397

[B32] White L. E. Andrews T. J. Hulette C. Richards A. Groelle M. Paydarfar J. (1997). Structure of the human sensorimotor system. I: morphology and cytoarchitecture of the central sulcus. *Cereb. Cortex* 7 18–30. 10.1093/cercor/7.1.18 9023429

[B33] Yousry T. A. Schmid U. D. Alkadhi H. Schmidt D. Peraud A. Buettner A. (1997). Localization of the motor hand area to a knob on the precentral gyrus. A new landmark. *Brain* 120 141–157. 10.1093/brain/120.1.141 9055804

